# Disruption of a C69-Family Cysteine Dipeptidase Gene Enhances Heat Shock and UV-B Tolerances in *Metarhizium acridum*

**DOI:** 10.3389/fmicb.2020.00849

**Published:** 2020-05-05

**Authors:** Juan Li, Mei Guo, Yueqing Cao, Yuxian Xia

**Affiliations:** ^1^School of Life Sciences, Chongqing University, Chongqing, China; ^2^Chongqing Engineering Research Center for Fungal Insecticides, Chongqing, China; ^3^Key Laboratory of Gene Function and Regulation Technologies, Chongqing Municipal Education Commission, Chongqing, China

**Keywords:** entomopathogenic fungi, dipeptidase, heat-shock tolerance, ultraviolet-B tolerance, C69 family

## Abstract

In fungi, peptidases play a crucial role in adaptability. At present, the roles of peptidases in ultraviolet (UV) and thermal tolerance are still unclear. In this study, a C69-family cysteine dipeptidase of the entomopathogenic fungus *Metarhizium acridum*, MaPepDA, was expressed in *Escherichia coli*. The purified enzyme had a molecular mass of 56-kDa, and displayed a high activity to dipeptide substrate with an optimal Ala-Gln hydrolytic activity at about pH 6.0 and 55°C. It was demonstrated that MaPepDA is an intracellular dipeptidase localized in the cytosol, and that it is expressed during the whole fungal growth. Disruption of the *MaPepDA* gene increased conidial germination, growth rate, and significantly improved the tolerance to UV-B and heat stress in *M. acridum*. However, virulence and conidia production was largely unaffected in the Δ*MaPepDA* mutant. Digital gene expression data revealed that the Δ*MaPepDA* mutant had a higher UV-B and heat-shock tolerance compared to wild type by regulating transcription of sets of genes involved in cell surface component, cell growth, DNA repair, amino acid metabolism, sugar metabolism and some important signaling pathways of stimulation. Our results suggested that disruption of the *MaPepDA* could potentially improve the performance of fungal pesticides in the field application with no adverse effect on virulence and conidiation.

## Introduction

The harmful and irreversible impacts of toxic chemical insecticides on the environment are receiving widespread attention and have accelerated extensive research for alternatives, especially biological control agents such as fungi and bacteria (Aw and Hue, 2017). *Metarhizium* is regarded as reliable substitute for chemical pesticide because of its distinctive advantages, such as safety, environmental friendliness, and low insect resistance (Steven et al., 2003; Jesper et al., 2007; Guerrero-Guerra et al., 2013).

*Metarhizium* can parasitize multiple insects, such as soil pests, leaf-feeding pests, and pests of cereal grains (Hu et al., 2014; Marion et al., 2016; Hong et al., 2017; Sahayaraj et al., 2018). Previous studies showed that *Metarhizium* had achieved good results in control of fruit fly (Yousef et al., 2017), locust (Peng et al., 2008; Brunner-Mendoza et al., 2019), and grasshopper (Milner et al., 2003). Compared with chemical pesticide and other fungal bioagents, such as *Beauveria bassiana*, and *Metarhizium rileyi*, *Metarhizium anisopliae* were more effective to *Helicoverpa armigera* infection in pigeon peas (Nahar et al., 2004). Combined application of *Metarhizium* with *Bacillus thuringiensis* was more effective in controlling pests than single use of *B. thuringiensis*, and the host insects could hardly develop resistance (Tupe et al., 2017). *Metarhizium* spores were reported to be detected in soil or as endophytes in plants root and can persist over a long time (Greenfield et al., 2016). However, many challenges, such as unstable tolerance to physical and natural conditions and low virulence, limited the efficiency and large-scale application of *Metarhizium*. Ultraviolet radiation and temperature fluctuations are two noticeable detrimental environmental factors affecting the viability of entomopathogenic fungi for pest control in field (Braga et al., 2015; Fernandes et al., 2015; Ortiz-Urquiza and Keyhani, 2015). Therefore, exploring the mechanism of heat shock and UV-B tolerance would help to improve the efficiency of fungal biocontrol agents by genetic engineering techniques (Zhao et al., 2016).

Peptidases (EC 3.4) are a class of enzymes to hydrolyze peptide chain to oligopeptides and single amino acids (Barrett, 1994). Peptidases can be classified into 261 families by catalytic mechanism as serine, cysteine, aspartyl, threonine, glutamic, or metallopeptidases (Rawlings and Bateman, 2019). For example, peptidases in which the nucleophile that attacks the scissile peptide bond is the sulfhydryl group of a cysteine residue are designated as cysteine-type peptidases (Rawlings et al., 2011; Barrett and Rawlingsm, 2013). The clans and families of cysteine peptidases were summarized in the review (Rawlings and Bateman, 2019).

Peptidases broadly exist in all organisms, playing a key role in the process of cell growth metabolism. Fungi produce extracellular peptidases to break down environmental protein and polypeptides and supply small molecules for fungal growth (St. Leger et al., 1997; James, 2006; Hamin Neto et al., 2018). The spectrum of these secreted peptidases were correlated with the fungal traits, making them possible markers of fungal ecology (Semenova et al., 2017). Serine proteases are essential for pathogenic fungi to utilize environmental nutrients and maintain their own reproduction (dos Santos et al., 2006). Extracellular peptidases play vital role in penetrating insect cuticle and contribute to pathogenicity of fungi (St. Leger, 1995; St. Leger et al., 1997; Yike, 2011; Semenova et al., 2020). Their vital roles in pathogenicity make them become markers of pathogenicity in fungi (Semenova et al., 2020). In human fungal pathogens, secreted peptidase increased the fungal survival and virulence in *Aspergillus fumigatus* (Beauvais et al., 1997), *Cryptococcus neoformans* (Clarke et al., 2016) and *Candida* (Dutton et al., 2016). In thermophilic fungi, peptidases improved their adaptations to high temperature, providing them with adequacy for biotechnological application (de Oliveira et al., 2018).

The dipeptidase (PepD) is an enzyme that cleaves dipeptides into two amino acids (EC 3.4.13). Up to now, few dipeptidases have been assayed and characterized. In *Saccharomyces cerevisiae*, Dug1p, a Cys-Gly-specific dipeptidase regulated the fungi-specific alternative pathway of glutathione degradation (Kaur et al., 2012). In human pathogenic fungus *Histoplasma capsulatum*, *N*-acetylated α-linked acidic dipeptidase performed as a major antigen during histoplasmosis (Toyotome et al., 2015). Dipeptidase A (PepDA) belongs to the cysteine peptidase family C69, which was evolutionarily conserved and found in 14 bacterial phyla and some eukaryotes. In fungi, the PepDA only existed in ascomycetes (Rawlings and Bateman, 2019). Family C69 dipeptidase was first characterized in *Lactobacillus farciminis*, showing an extremely strict substrate specificity (Sakamoto et al., 2013). Functional analysis revealed that the *PepDA* did not affect the growth in *Lactobacillus helveticus* (Dudley et al., 1996). Up to now, the functions and enzyme characteristics of C69 family dipeptidase are rarely understood in filamentous fungi.

In this study, we performed experiments to characterize the function of a C69 family dipeptidase gene *MaPepDA* in some important biocontrol traits by gene disruption in *M. acridum*. We found that MaPepDA was localized in cytoplasm. Disruption of the *MaPepDA* gene increased conidial germination, UV-B tolerance and heat-shock tolerance in *M. acridum*. Digital gene expression profiling (DGE) results showed that the *MaPepDA* affected the transcription of genes involved in cell surface component, cell growth, DNA repair, amino acid metabolism and sugar metabolism. Exceptionally, virulence was largely unaffected in Δ*MaPepDA* mutant.

## Materials and Methods

### Strains and Growth Conditions

The fungal wild-type (WT) strain of *M. acridum* CQMa102 was stored in the China General Microbiological Culture Collection Center (CGMCC; No. 0877). The mutant Δ*MaPepDA* described in this study was constructed by disruption of the *MaPepDA* gene from wild-type strain. All fungal strains used in this study were grown on one-quarter-strength Sabouraud’s dextrose agar medium (1/4 SDAY consisting of 1% dextrose, 0.25% mycological peptone, 0.5% yeast extract, and 2% agar, w/v) at 28°C for 15 days to obtain mature conidia unless specifically states. The *Escherichia coli* strains DH5 and BL21 (TransGen Biotech, Beijing, China) were used for cloning experiments and protein expression, respectively. The bacterial strains were cultured at 37°C in Luria-Bertani (LB) with vigorous shaking. *Agrobacterium tumefaciens* AGL-1 was used for fungal transformations. The plasmid pET-28a (+) (Novagen, Madison, WI, United States) was used for protein expression.

### Molecular Cloning and Phylogenetic Analysis

Genomic DNAs were isolated from mycelium using DNA Extraction Kit (OMEGA, Georgia, United States). Fungal total RNAs were extracted from mycelia using RNA Extraction Kit (CWBIO, Beijing, China). Complementary DNA (cDNA) synthesis was performed following the manufacturer’s instructions of the PrimeScript^TM^ RT reagent Kit with gDNA Eraser (Perfect Real Time) Kit (TaKaRa, Dalian, China). The whole cDNA sequence of *MaPepDA* was amplified using primers MaPepD-CF and MaPepD-CR (Supplementary Table S1). The pMD19-T vector was used for cDNA sequencing (TaKaRa, Dalian, China).

### Gene Disruption and Complementation

DNA cassettes for the targeted *MaPepDA* gene disruption were generated using homologous recombination technique. The disruption vector pK2-PB-MaPepD was including a 1.1-kb 5′-flanking sequence and a 0.8-kb 3′-flanking sequence of the *MaPepDA* gene. The two fragments were amplified with LF/LR and RF/RR (Supplementary Table S1) and then inserted into the pK2-PB vector (Cao et al., 2014). The pK2-bar-MaPepD was transformed into WT *M. acridum* via *Agrobacterium tumefaciens*-mediated transformation, and transformants were screened according to previously described method (dos Reis et al., 2004). Two primer pairs of MaPepD-VF/LB-PT-R (Supplementary Table S1) and RB-Bar-F/MaPepD-VR (Supplementary Table S1) were used to verify the correct insertion of pK2-PB-MaPepD. The complementation vector pK2-sur-MaPepD:Enhanced green fluorescence protein (EGFP) contained a 3.5-kb fragment including the 1.5-kb *MaPepDA* gene coding sequence and the 2.0-kb promoter region, which was amplified with primer pair MaPepD-CP-F/MaPepD-CP-R (Supplementary Table S1). The pK_2_-sur-MaPepD:egfp vector were transformed into Δ*MaPepDA*. Transformants were screened on Czapek-Dox medium containing 20 μg/ml chorimuronethyl (Sigma, Bellefonte, PA, United States) and confirmed by Southern blotting. Probe preparation (PF/PR) and blotting was performed according to the instructions of High Prime DNA Labeling and Detection Starter Kit I (Roche, Mannheim, Germany).

### Protein Subcellular Localization and Gene Transcription

The subcellular localization of MaPepDA was determined in complemented strains (CP) in which the *MaPepDA* was fused with an *egfp* gene. Total RNAs were extracted from wild strain culture after growing 1, 3, 6, 9, 12, 15 day on 1/4 SDAY. Transcription level of the *MaPepDA* gene was determined by qRT-PCR with primer QF/QR (Supplementary Table S1) using a SYRB Premix Ex Taq^TM^ II kit (TaKaRa, Dalian, China) according to the manufacturer’s instructions. The method of 2-^(ΔΔCt)^ was employed to calculate the relative gene expression levels (Livak and Schmittgen, 2001). The glyceraldehyde-3-phosphate dehydrogenase gene *Magpd* (gpdF/gpdR, EFY84384) was employed to normalize the amount of template cDNA in each reaction. Data were expressed as the mean ± SE (standard error) of three independent experiments.

### Growth, Germination, and Conidiation

Conidial suspensions (50 μl) at a concentration of 1 × 10^7^ conidia/ml were spread evenly on 1/4 SDAY plates, and the plates were incubated at 28°C. Germinated spores were counted every 2 h until control spores germinated almost completely. Percent germination indicated the percentage of germinated spores in a total of 100 randomly selected spores. A spore was considered as germinated when the length of germ tube reached one half of its width (Cao et al., 2014). The determination was conducted in triplicate. The mean 50% germination time (GT50) was then calculated. Hyphal growth was examined 16 h post inoculation and photographed under a microscope (Nikon Eclipse Ci-E, Tokyo, Japan). To observe colony morphology, 2 μl aliquots of conidial suspension (1 × 10^6^ conidia/ml) was dropped on 1/4 SDAY and the plates were incubated at 28°C for 5 days. Conidia production was determined on 1/4 SDAY as described previously (Cao et al., 2014).

### Tolerance to Stresses

Fungal conidia suspensions were dotted on 1/4 SDAY supplemented with the following stressors: 0.01% sodium dodecyl sulfate (SDS), 200 μg/ml Calcofluor White (CFW), 500 μg/ml congo red, and 6 mmol/l H_2_O_2_. Sensitivity to each chemical was represented by relative growth inhibition (RGI), which was determined with the equation [(Dc − Dt)/Dc × 100], where Dc and Dt indicate the colony diameter of fungal strains under control and stressed conditions, respectively. Tolerance to heat and ultraviolet-B irradiation (1350 mW/m^2^) was determined according to previous reports (Rangel et al., 2005; Liu et al., 2010). Following UV and heat treatment, the conidial germination was evaluated after growing 20 h on 1/4 SDAY. IT_50_ (time for 50% inhibition in germination rate by heat or UV irradiation) was calculated with the program GraphPad Prism and compared among WT, Δ*MaPepDA*, and CP strains.

### Bioassays

Bioassay was conducted against fifth-instar nymphs of *L. migratoria manilensis* (Meyen) in two ways. Aliquots (3 μl) of conidial suspensions from WT, Δ*MaPepDA*, and CP strains were applied topically on the pronotum at a concentration of 1 × 10^7^ conidia/ml in paraffin oil or injected into the hemolymph of locusts at 1 × 10^6^ conidia/ml in sterile water. Three groups of 30 locusts for each bioassay were used for each strain. The locusts were fed on fresh corn leaves at 28°C and 75% relative humidity with a 12:12 h light:dark photoperiod. The dead locusts were removed and the number of dead locusts was recorded every 12 h until all the locusts infected with fungus died. Mean median lethal time (LT_50_) was estimated using the program GraphPad Prism 8.0 and compared among the wild-type, Δ*MaPepDA*, and CP strains. The experiments were conducted three times.

### Expression and Purification of Recombinant MaPepDA

To analyze the enzyme characteristics of MaPepDA, the *MaPepDA* was expressed in pET-28a carrying a His-tag. To construct the recombinant plasmids of pET-28a-MaPepD-His, The *MaPepDA* cDNA sequence was amplified with Eo-F/Eo-R (Supplementary Table S1) and inserted into *Nco*I/*Xho*I-digested pET-28a (+) vector. The recombinant plasmids amplified in *E. coli* DH5α were transformed in *E. coli* BL21, and the transformants were screened and cultured on an LB plate supplemented with 50 μg/ml kanamycin at 37°C overnight. Single colonies were inoculated into LB containing 50 μg/mL kanamycin, and shaken at 37°C for 12 h. This culture (1%, v/v) was then inoculated into 200 ml of LB liquid medium, grew at 37°C for 6 h with 200 rpm and then 500 μl isopropylthio–galactoside (IPTG, 500 mM) was added for induction at 18°C for 20 h. Cells were harvested at 12,000 rpm for 2 min at 4°C, washed twice with 10 ml of lysis buffer (10 mM HEPES, 10 mM NaCl; pH 7.5). The cells were broken by ultrasonic, and then the lysate was centrifuged at 10,000 rpm for 10 min at 4°C. The soluble fraction was analyzed by SDS-Polyacrylamide Gel Electrophoresis (SDS-PAGE).

The recombinant MaPepDA protein (rMaPepDA) was purified using an immobilized nickel-nitrilotriacetic acid (Ni-NTA) affinity column (GE Healthcare Life Science, Marlborough, United States) with AKTA prime plus (GE Healthcare Life Science, Uppsala, Sweden) (Seo et al., 2007). The purified rMaPepD was analyzed by SDS-PAGE.

### Substrate Specificity and Activity Assay

Substrate specificity of the MaPepDA enzyme was investigated using the cadmium-ninhydrin (Cd-ninhydrin) assay to analyze the hydrolysis of dipeptides (Folkertsma and Fox, 1992). Three dipeptide substrates, Ala-Gln, Gly-Pro, and Leu-Phe were used in substrate specificity and enzyme activity assay. Enzyme solution 50 μl and 50 μl of substrates (25 mM in ddH_2_O) were mixed with 400 μl of reaction buffer (50 mM Tris-HCl buffer, pH 8.0). The reaction mixture was incubated at 37°C for 0 min, 10 min, 30 min, 40 min, and 60 min, respectively. The reaction was terminated by adding 1 ml of Cd-ninhydrin reagent. The mixture was heated at 84°C for 5 min and cooled immediately on ice. The absorbance at 485 nm was measured (Elsoda et al., 1992). One unit of enzyme activity was defined as the amount of enzyme that increased the absorbance 0.01 per minute under the assay conditions. The protein concentration was measured by Bradford protein assay method with bovine serum albumin (Sigma-Aldrich, CA, United States) as the standard. The absorbance at 595 nm was determined with microplate reader (Berthold Technologies, Germany).

To estimate the kinetic parameters of the rMaPepDA, substrates (Ala-Gln, Gly-Pro, and Leu-Phe) were used at concentrations ranging from 12.5–75 mM. Lineweaver-Burk plots (Gobbetti and Fox, 1998) and a Hanes transformation (Hanes, 1983) was used to calculate the Michaelis–Menten constant (*K*_m_) of the MaPepDA enzyme. Substrates at different concentrations without of MaPepDA enzyme were used as controls. The reaction of mixtures was assayed as described above.

### Optimal pH and Temperature of rMaPepDA

In order to elucidate the influence of pH on enzymatic activity, the rMaPepDA activity was assayed against Ala-Gln in the pH range from 4.0 to 8.0, using the following buffers: maleic acid-NaOH (pH 4.0 to 6.0), PBS (pH 7.0), Tris-HCl (8.0). Reaction mixtures were incubated at 37°C for 30 min. The optimal temperature was determined by analyzing dipeptidase activity at temperatures ranging from 25°C to 90°C in 50 mM Tris-HCl buffer (pH 8.0). The residual activity was subsequently measured with Ala-Gln as the substrate at pH 8.0 (Sakamoto et al., 2013). The relative activity was defined as the percentage of activity comparing to the highest activity.

### Digital Gene Expression Profiling (DGE)

The total RNAs were extracted from 3-day culture of Δ*MaPepDA* and WT on 1/4 SDAY plate. DGE sequencing was performed using the Illumina HiSeq 2000 at the Beijing Genomics Institute (BGI) (Wuhan, China). Genes with a false discovery rate (FDR) < 0.001 and more than a two-fold change were regarded as differentially expressed genes (DEGs) (Audic and Claverie, 1997). DEGs were classified and annotated using Gene Ontology (GO) analysis and Kyoto Encyclopedia of Genes and Genomes (KEGG) pathway enrichment analysis. To further verify the results of DGE, transcription of 24 differential expressed genes were determined by qRT-PCR with primers listed in Supplementary Table S2.

### Statistical Analysis

The ANOVA one-way model with Date processing System program 22.0 (IBM SPSS Statistical, Chicago, IL, United States) was used to analyze percent germination, conidia yield, GT_50_, IT_50_, RIG, and LT_50_. Tukey’s honest significant difference test was used to separate means at α = 0.05 or 0.01.

## Results

### MaPepDA Is Conserved Among Fungi

**FIGURE 1:**
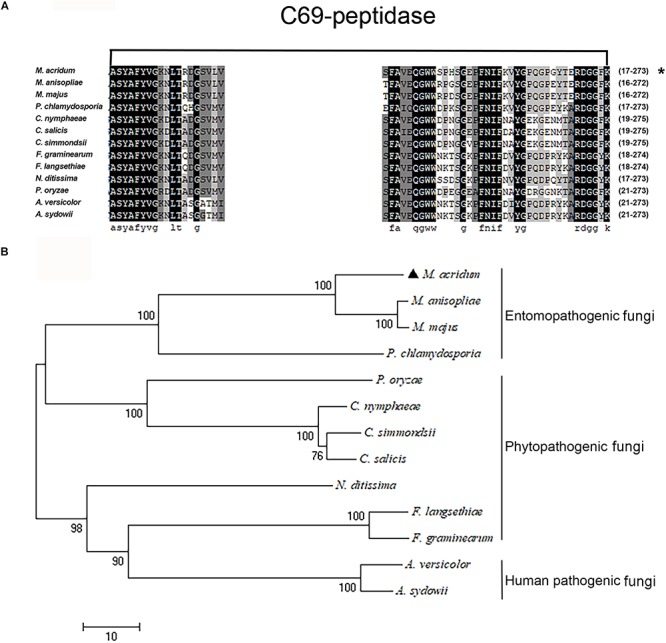
Bioinformatics analysis of the MaPepDA protein. **(A)** MaPepDA shared conserved domain with other C69 dipeptidase homologs. **(B)** Phylogenetic analysis of PepDA family proteins from the selected human, plant, and insect fungal pathogens. *M. acridum*: *Metarhizium acridum* (XP_007815714); *M. majus*: *Metarhizium majus* (KID96910); *M. anisopliae*: *Metarhizium anisopliae* (KFG80018); *P. chlamydosporia*: *Pochonia chlamydosporia* (XP_018139060); *P. oryzae*: *Pyricularia oryzae* (ELQ32800); *C. nymphaeae*: *Colletotrichum nymphaeae* (KXH34606); *C. simmondsii*: *Colletotrichum simmondsii* (KXH29269); *C. salicis*: *Colletotrichum salicis.* (KXH50308); *N. ditissima*: *Neonectria ditissima* (KPM38691); *F. graminearum*: *Fusarium graminearum* (XP_011328638); *F. langsethiae*: *Fusarium langsethiae* (KPA42066); *A. sydowii*: *Aspergillus sydowii* (OJJ60401); *A. versicolor*: *Aspergillus versicolor* (OJI99730). The full amino acid sequences were aligned with Clustal X and a neighbor joining tree was generated with 1,000 boot strap replicates using the program MEGA v4.0 (Tamura et al., 2007). *means the sequence of MaPepDA.

The protein sequence of MaPepDA (XP_007815714) had 521 amino acids with a predicted molecular weight 57 kDa. The multiple sequence alignment analysis showed that the C69 domain of MaPepDA was homologous to C69 family peptidases of other fungal species (Figure 1A). Phylogenetic analysis revealed that MaPepDA was clustered with other PepDA homologs from entomopathogenic fungi (Figure 1B).

**FIGURE 2:**
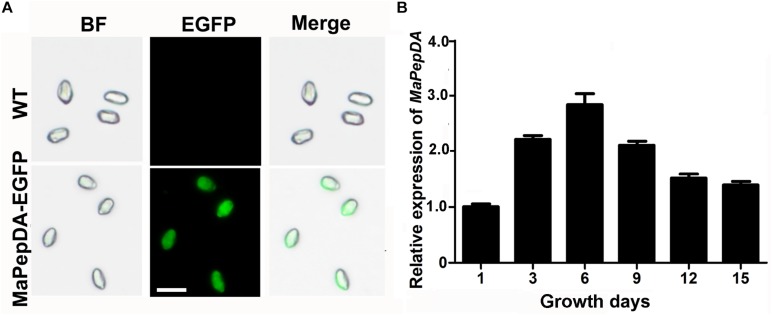
Subcellular localization of MaPepDA and the transcription of *MaPepDA* in *M. acridum*. **(A)** Subcellular localization of MaPepDA. BF, bright field. Bars indicate 5 μm. **(B)** Transcription of *MaPepDA* in different growth period. The transcription level of *MaPepDA* on the first growth day was designed as 1 and transcription on other days were compared with the first growth day.

In order to analyze the cellular localization of MaPepDA, EGFP-tagged MaPepDA fusion protein under regulation of the native promoter was expressed in Δ*MaPepDA* mutant. Fluorescent microscopic visualization showed that the fusion MaPepDA-EGFP was evenly localized in cytoplasm (Figure 2A), which was consistent with the estimated results of the online program^1^. Time-course analysis of transcription showed that the *MaPepDA* was expressed during the whole fungal growth (Figure 2B).

### Purification and Characterization of rMaPepDA

**FIGURE 3:**
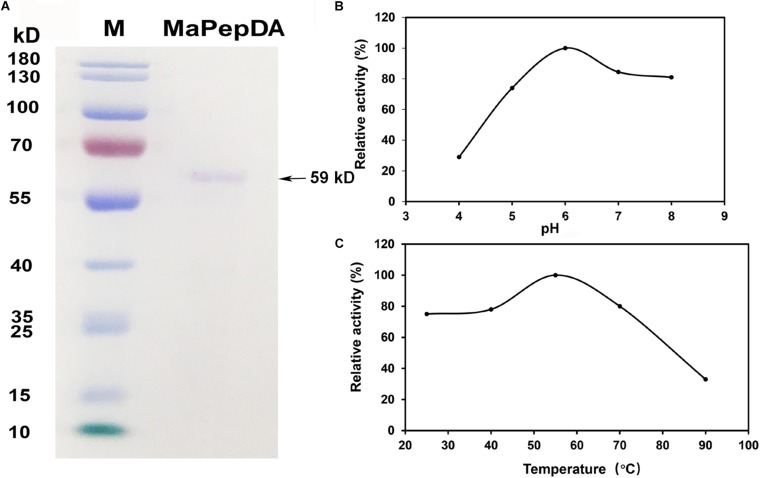
Expression, purification and activity assay of rMaPepDA. **(A)** SDS-PAGE analysis of purified rMaPepDA. **(B)** Effects of pH on rMaPepDA activity. **(C)** Effects of temperature on rMaPepDA activity. The dipeptide of Ala-Gln was acted as the substrate in activity analysis. The assay was performed with three replicates.

The MaPepDA protein was induced with 1 mM IPTG and further purified using a Ni-chelating resin column. Results showed that MaPepDA was successfully expressed in *E. coli* BL21. SDS-PAGE showed that MaPepDA-His had a molecular mass of about 59-kDa, which was close to the theoretical molecular mass (56 kDa) deduced from the MaPepDA sequence (Figure 3A). The MaPepDA showed high activity when the pH was in the range 6.0 to 8.0 (≥74%) with maximal activity at pH 6.0. Compared with the activity at pH 6.0, the activity of MaPepDA decreased about 70% when pH was 4.0 (Figure 3B). MaPepDA showed a wide thermal resistance ranging from 25°C and 70°C. MaPepDA enzyme activity exhibited about 75% and 80% of the maximum at 25°C and 70°C, respectively, but decreased rapidly with the increasing temperature up to 90°C (Figure 3C).

To explore the substrate specificity and activity of MaPepDA, the purified enzyme solution was incubated with different substrates, Ala-Gln, Leu-Phe, and Gly-Pro, at various concentrations. Enzyme activity of MaPepDA was quantified using the Cd-ninhydrin assay after the hydrolysis of dipeptides. Judging from *K*_m_ values in Table 1, MaPepDA had a higher substrate affinity for Leu-Phe (186 mM), followed by Gly-Pro (768 mM) and Ala-Gln (1645 mM). Substrate specificity analysis showed that MaPepDA had 240 U/mg activity toward Ala-Gln, 132 U/mg toward Leu-Phe, and 47 U/mg toward Gly-Pro (Table 1).

**TABLE 1:** Substrate specificity and kinetic parameter of MaPepDA.

**Substrate**	**Enzyme activity (U/mg)**	**Relative activity**	***K*_m_ (mM)**
Ala-Gln	240	100%	1645
Leu-Phe	132	54%	186
Gly-Pro	47	19%	768

### Deletion and Complementation of the *MaPepDA* Gene

In order to analyze the functions of *MaPepDA* in *M. acridum*, the Δ*MaPepDA* mutant was generated by homologous recombination, in which the *MaPepDA* open reading frame (ORF) was replaced by a bar gene (Supplementary Figure S1A).

The *MaPepDA* gene was complemented by random inserting a functional copy. The complementation plasmid was carrying MaPepDA native promoter and an EGFP fusion gene (Supplementary Figure S1A). PCR (Supplementary Figure S1B) and Southern blot (Supplementary Figure S1C) confirmed that the Δ*MaPepDA* strain had correctly inserted the *Bar* disruption cassette. The hybridizing band was about 2.6-kb in WT, 3.3-kb in mutant, and 2.6 and 3.3-kb bands both appeared in CP strain, indicating that the *MaPepDA* gene was targeted disrupted (Supplementary Figure S1C).

### MaPepDA Had a Crucial Role in Conidial Germination and Hyphae Growth

**FIGURE 4:**
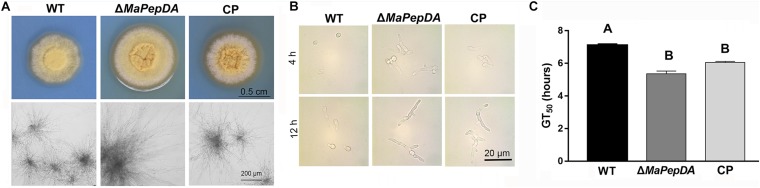
The *MaPepDA* was essential for conidial germination and hyphae polarized growth. **(A)** Colony morphology and hyphae growth on 1/4 SDAY. **(B)** Germination of three strains at 4 h and 12 h post inoculation. **(C)** The mean 50% germination time (GT_50_) of fungal strains. Different capital letters denote significant differences at *p* < 0.01.

Growth on plate showed that the colony diameter of the Δ*MaPepDA* mutant obviously increased compared with WT and CP (Figure 4A). Δ*MaPepDA* mutant had much longer and more densely aerial hyphae compared with the WT (Figure 4A).

The conidia of Δ*MaPepDA* mutant germinated at 4 h, while most WT and CP conidia were still intact, suggesting an accelerated conidial germination in Δ*MaPepDA* compared to WT and CP strains (Figure 4B). The mean 50% germination time (GT_50_) of Δ*MaPepDA* (5.35 ± 0.20 h) was significantly decreased compared to WT (7.10 ± 0.06 h) (*P* < 0.01, Figure 4C). However, time-course analysis of conidia production showed that MaPepDA didn’t affect conidia yield in *M. acridum* (Supplementary Figure S2).

### Disruption of the *MaPepDA* Gene Enhanced Heat-Shock Tolerance

**FIGURE 5:**
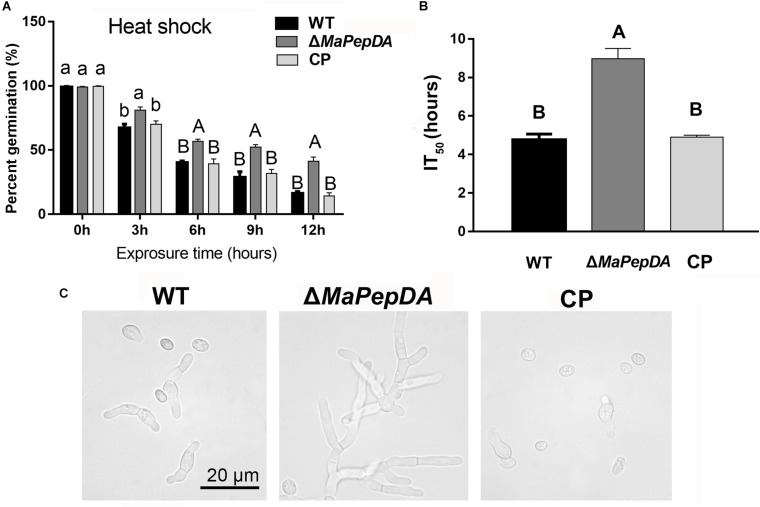
The *MaPepDA* negatively controlled fungal tolerances to heat shock. **(A)** Conidial germination was counted after exposure to 45°C for 3, 6, 9, and 12 h. All strains were then cultured on 1/4 SDAY medium at 28°C for 20 h. **(B)** The mean 50% inhibition time (IT_50_) under heat shock. Different capital letters denote significant differences at *p* < 0.01. **(C)** Conidial germination was photographed after exposure to 45°C for 6 h, followed by culturing at 28°C for 20 h.

In order to estimate the survival under natural stress, the fungal tolerance to heat-shock was analyzed. A time-course study of heat-treatment showed that the conidial germination of WT was significantly lower than Δ*MaPepDA* (*P* < 0.01; Figure 5A). The mean 50% inhibition time (IT_50_) of Δ*MaPepDA* was 8.98 ± 0.99 h, significantly increased compared to WT and CP, which were 4.76 ± 0.54 h and 4.89 ± 0.19 h, respectively (*P* < 0.01; Figure 5B).

The fungal germination was examined under microscope for the 6-h treatment group. Figure 5C showed that a substantial number of spores the Δ*MaPepDA* conidia germinated and produced long hyphae, while most WT and CP conidia still do not have a discernible germ tube. These results suggested that disruption of the *MaPepDA* increased tolerance to heat shock in *M. acridum.*

### MaPepDA Negatively Regulates UV-B Tolerance

**FIGURE 6:**
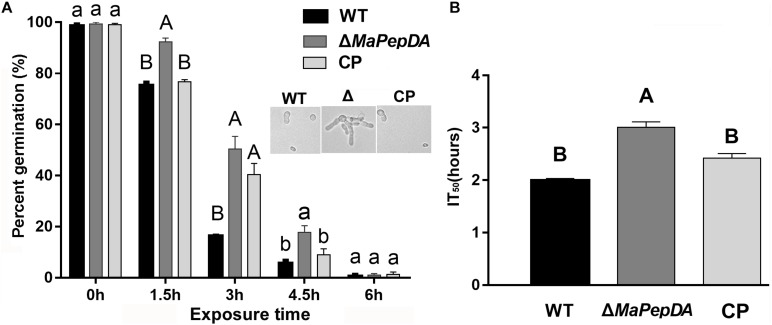
The *MaPepDA* negatively regulated tolerance to UV-B irradiation. **(A)** Conidial germination rate was counted after exposure to UV-B irradiation (1350 mW/m^2^) for 1.5, 3, 4.5, and 6 h. Upper diagram in panel A showed the conidial germination after exposure to UV-B irradiation for 4.5 h. All strains were then cultured on 1/4 SDAY medium at 28°C for 20 h. **(B)** IT_50_ under UV-B irradiation. Capitalized letter: significant difference, *p* < 0.01; lowercase letter: significant difference, *p* < 0.05.

Ultraviolet radiation is one of the most important factors influencing the conidia survival. Therefore, the UV-B tolerance was evaluated in strains of Δ*MaPepDA*, WT and CP. After exposed to UV-B irradiation for a period of time, conidial germination was analyzed after growing for 20 h. The Δ*MaPepDA* mutant had 40% and 18% germination after 3 h and 4.5 h of UV-B treatment, respectively, which was significantly higher than that of WT (17% and 8%, respectively) (*P* < 0.01, Figure 6A). Microscope observation of conidial and hyphal morphology at 4.5 h showed that WT and CP strains had tiny germ tubes, while Δ*MaPepDA* conidia generated much longer hyphae (Figure 6A). The IT_50_ of Δ*MaPepDA* was 3.00 ± 0.20 h, significantly increased compared to WT and CP, which were 1.99 ± 0.05 h and 2.41 ± 0.14 h, respectively (*P* < 0.01; Figure 6B). These results suggested that the *MaPepDA* gene negatively affected the tolerance to UV-B stress.

### The *MaPepDA* Affects the Tolerance of Cell Wall-Disturbing Agents

**FIGURE 7:**
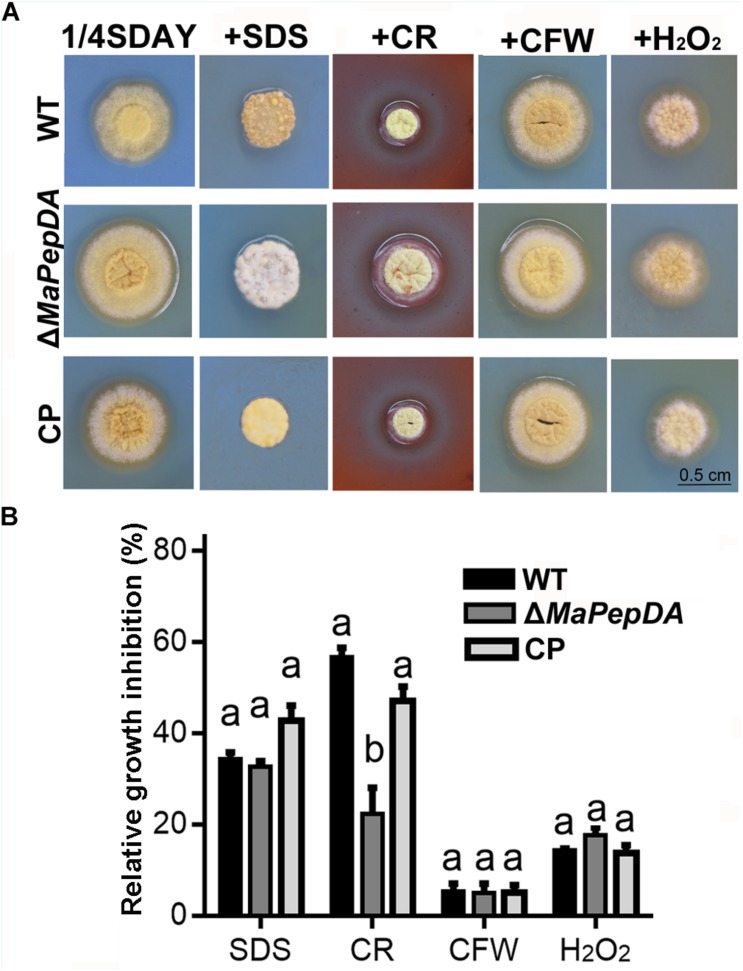
Phenotypic characterization of different fungal strains. **(A)** Strains grew on 1/4 SDAY medium or 1/4 SDAY medium supplemented with 50 mg CFW, 500 mg CR or 6 mmol H_2_O_2_ at 28°C for 6 days. **(B)** Relative inhibition growth of chemical stressors. Different lowercase letters denote significant differences at *p* < 0.05.

Spot assays were performed to investigate the sensitivity of fungal strains to cell wall disturbing agents (Figure 7A). Δ*MaPepDA* had 22.3% RGI when CR was included in the medium, significantly lower than WT and CP strains (*P* < 0.05), which were 56.7% and 47.1%, respectively (Figure 7B). However, the RGI of Δ*MaPepDA* had no significant difference with WT and CP under SDS, H_2_O_2_ and CFW stressors (*P* > 0.05, Figure 7B).

### *MaPepDA* Had No Effect on Virulence in *M. acridum*

To evaluate the contribution of the *MaPepDA* to virulence in *M. acridum*, we conducted pathogenicity assay by applying conidia topically on pronotum (Supplementary Figure S3A) or injected (Supplementary Figure S3B) into the hemolymph of *L. migratoria manilensis* with conidia suspensions of WT, Δ*MaPepDA* and CP strains. Results showed that the Δ*MaPepDA* did not affect virulence in *M. acridum.* There was no difference in LT_50_ among WT, Δ*MaPepDA*, and CP strains both in topical assay (*P* > 0.05, Supplementary Figure S3C) or injection assay (*P* > 0.05, Supplementary Figure S3D).

### Identification of DEGs Regulated by the *MaPepDA*

**FIGURE 8:**
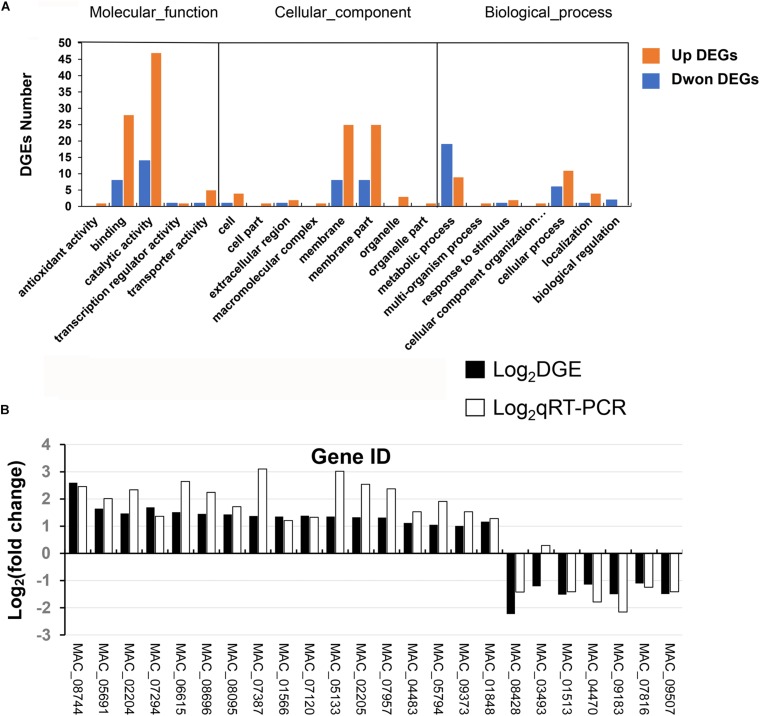
RNA_seq analysis. **(A)** GO annotation of differentially expressed genes. **(B)** qRT-PCR analysis to verify RNA_seq results.

In order to further explore the mechanism of the *MaPepDA* gene in regulating the stress resistance and growth, RNA-seq was performed to compare the differentially expressed genes between Δ*MaPepDA* and WT. RNA-seq analysis mapped 9761 transcripts of the *M. acridum* genes. Differential expression analysis identified 132 transcripts with significant expression changes (*p* ≤ 0.05 and fold change ≥ 2). RNA-seq revealed that 98 transcripts were commonly up-regulated and 34 transcripts were down-regulated. Gene ontology (GO) based enrichment analysis was carried out using a threshold value (*p* ≤ 0.05). GO annotation suggested that the DEGs were divided into three categories with 106 in molecular function, 54 in biological process, and 80 in cellular component (Figure 8A). The classification results (Supplementary Table S3) showed that these functional differential genes were mainly involved in multiple life processes such as growth (20 genes), sporulation (3 genes), cell wall components (11 genes), DNA damage repair (4 genes), stress tolerance (12 genes), amino acid metabolism (10 genes), sugar metabolism and transportation (5 genes), and some important signaling pathways.

In order to analyze the reliability of digital expression profiling, 24 DEGs were selected for qRT-PCR analysis to verify the RNA-seq results. Twenty-three genes showed similar expression patterns in both qRT-PCR and RNA-seq analysis except for one gene (MAC_03493), indicating that the RNA-seq data was reliable (Figure 8B).

## Discussion

At present, the research of dipeptidase mainly focuses on animal (O’Dwyer et al., 2009; Andreyeva et al., 2019; Choudhury et al., 2019), human pathogenic bacteria (Chang et al., 2010; Toyotome et al., 2015) or plant pathogenic bacteria (Kumar et al., 2014). Function reports of dipeptidases are mostly limited to virulence and growth in microorganisms, while there is little information regarding the effect of dipeptidases on stress tolerance in microorganisms. Family C69 dipeptidase is strictly distributed in fungi to ascomycetes (Rawlings and Bateman, 2019). Few studies were reported on PepDA in microorganisms and no functional analysis has been reported in fungi.

Peptidases produced by periodontopathic bacteria provide nutrients for bacterial growth, and are important etiologic reagents involved in degradation of periodontal tissues and alterations of the host immune system and bioactive peptides (Potempa et al., 2000). Our biochemical data confirmed that the MaPepDA had broad substrate specificity and could efficiently hydrolyze dipeptides to single amino acid, even toward the hard-to-degrade Gly-Pro sequence. This characteristic of Gly-Pro degrading is also rare for bacterial dipeptidase (Sakamoto et al., 2013). Consistent with bacterial PepDA (Sakamoto et al., 2013), MaPepDA had a high affinity to the substrate Ala-Gln and a strong tolerance to high temperature. MaPepDA has similar enzymological characteristics as in bacteria, indicating that PepDA was conservative in biochemical properties in different microorganisms.

During the infection process, entomopathogenic fungi secret many proteinases to degrade the insect cuticle. Previous reports showed that peptidases were mostly secreted outside and some peptidases played certain roles in virulence in entomopathogenic fungi (St. Leger, 1995; St. Leger et al., 1997). However, there is no report about the function of dipeptidases on virulence of pathogens. Our study showed that *MaPepDA* did not affect virulence in *M. acridum*. As an intracellular peptidase, MaPepDA might diverge in function in virulence due to the different subcellular location from other peptidases. MaPepDA-EGFP fusion expression showed that MaPepDA was diffused in cytoplasm and expressed during the whole fungal growth, suggesting a role of MaPepDA in growth. Consistent with the expression results, fungal growth analysis demonstrated that MaPepDA negatively regulated fungal growth. Also, RNA_seq results showed that many genes related amino acid metabolism were up-regulated (Supplementary Table S2) in Δ*MaPepDA*, which might balance the changes of protein metabolism when MaPepDA was inactivated. For example, carboxypeptidase (MAC_08739), a similar function as the virulence factor Pr1 (St. Leger et al., 1994), was upregulated in Δ*MaPepDA*.

Unlike no influence of PepDA on growth in bacteria (Sakamoto et al., 2013), Δ*MaPepDA* mutant had an increased growth and earlier conidial germination compared to WT strain, suggesting a functional diversity of PepDA in different organisms. Consistent with the changes of phenotype, RNA-seq data showed that some genes related to fungal growth and germination were up-regulated in Δ*MaPepDA.* SpoC1-C1C (MAC_08744), a conidial specific transcriptional activator, was up-regulated in Δ*MaPepDA*, which was reported highly expressed during the conidial germination and the mycelial growth in *Aspergillus nidulans* (Stephens et al., 1999). Fatty acid metabolism-related gene 3,2-*trans-*enoyl-CoA isomerase precursor (MAC_05794), which affected the growth in yeast (Gurvitz et al., 1998), had doubled transcription when the *MaPepDA* was impaired. Up-regulated cytochrome P450 genes (MAC_07120, MAC_07845) in Δ*MaPepDA* were reported to contribute to polarized growth and conidiogenesis in *Fusarium graminearum* (Shin et al., 2017). Nine of ten DEGs related to amino acid metabolism were up-regulated when the *MaPepDA* was disrupted, which was consistent with the more active growth in Δ*MaPepDA.*

When applied in the field, the two detrimental environmental stresses, heat and UV-irradiation, can kill the conidia, which limits the ability of fungi to survive and infect hosts in the environment, and also affect the proliferation and dispersion of fungi (Fernandes et al., 2015; Ortiz-Urquiza and Keyhani, 2015; Rangel et al., 2015). Our study showed that disruption of the *MaPepDA* gene significantly improved the fungal resistance to UV-B, heat-shock and chemical stresses. Transcription data suggested that disruption of the *MaPepDA* triggered up-regulation of some genes related to different stress tolerance, such as genes involved in DNA damage repair and cell wall proteins. Indole-diterpene biosynthesis protein PaxU (MAC_01848) was defined as a DNA repair protein RAD57 in KEGG (K10958), which was required for genetic recombination and DNA repair (Sung, 1997). A/G-specific adenine glycosylase (MAC_09373) was reported to be involved in DNA damage repair processes, repairing A/G in DNA and A/8-oxoG mismatches in *E. coli* (Lu, 2000). MAC_09373 was up-regulated in a protein phosphatase gene *MaPpt* mutant in *M. acridum*. Similar as *MaPepDA*, the *MaPpt* gene is also a negative regulator of UV stress tolerance (Zhang et al., 2019). Aspartate aminotransferase (MAC_08901), defined as a Xeroderma pigmentosum group C-complementing protein in KEGG (K10958), was involved in the main process of removing UV damage and many chemical lesions from DNA (Jones and Wood, 1993). In yeast, the cysteine-rich protein (MAC_07294) activated the transcription of metallothionein genes (MT) (Szczypka and Thiele, 1989), which bound heavy metal, scavenged free radicals, and protected DNA from radiation damage (Jie et al., 2005). In plant cell, the transcription level of 4-coumarate-CoA ligase (MAC_07387) was increased after UV irradiation, playing a crucial role in protection from UV radiation (Kuhn et al., 1984; Mierziak et al., 2014).

Cell wall proteins are involved in fungal cell wall integrity and are also critical for stress tolerance. The cell wall protein ecm33 mutant displayed hypersensitivity to temperature in *Candida albicans* (Gil-Bona et al., 2016). Our results demonstrated that two cell wall proteins (MAC_02204, MAC_05133) were up-regulated in Δ*MaPepDA*, possibly beneficial for the increased stress tolerance of the mutant strain. Glycosylphosphatidylinositol metabolism was involved in heat tolerance in *S. cerevisiae* (Nasution et al., 2015). Inositol monophosphatase (MAC_04535) played a key role in the phosphatidylinositol signaling pathway (Goswami et al., 2018). Up-regulation of inositol monophosphatase might contribute to the increased heat tolerance in *MaPepDA* null mutant. Chitinases, up-regulated in Δ*MaPepDA* (MAC_02205), have also displayed important roles in thermal stress tolerance in yeast (Versele and Thevelein, 2001) and *M. anisopliae* (Staats et al., 2013).

The RTA1 domain protein (MAC_01513) and catalase (MAC_04470) genes were down-regulated in Δ*MaPepDA* (Supplementary Table S2). In yeast, the cysteine-rich protein activated the transcription of metallothionein genes (MT) (Szczypka and Thiele, 1989), which bound heavy metal, scavenged free radicals, and protected DNA from radiation damage, thereby increasing the strain’s UV-B tolerance (Jie et al., 2005). In *S. cerevisiae*, the RTA1 domain protein was involved in the regulation of strain resistance, which led to strong resistance of *S. cerevisiae* to inhibitors and stimulation (Soustre et al., 1996).

## Conclusion

In conclusion, our results showed the characteristics of a typical C69-family cysteine dipeptidase in *M. acridum*. Biological function analysis demonstrated that disruption of the *MaPepDA* resulted in increased conidial germination, growth rate, and significantly improved the tolerance to UV-B and thermal stress compared to WT. Since UV and thermal stress susceptibilities represent the important potential limitation to the practical application of fungal bio-agents in field, further manipulations, such as engineered techniques, may be attempted on PepDA to potentially improve the efficiency in field application of entomopathogenic fungi.

## Data Availability Statement

The datasets generated for this study can be found in the PRJNA540381.

## Author Contributions

JL conducted the main experiment and wrote the manuscript. MG conducted RNA transcription. JL and YC analyzed the data. YX and YC conceived and designed the experiments. YX and YC provided technical oversight and critical manuscript review and editing.

## Conflict of Interest

The authors declare that the research was conducted in the absence of any commercial or financial relationships that could be construed as a potential conflict of interest.

## Footnotes

1 http://www.csbio.sjtu.edu.cn/bioinf/Cell-PLoc-2/
