# Supplementary material for: Disruption of a C69-Family Cysteine Dipeptidase Gene Enhances Heat Shock and UV-B Tolerances in Metarhizium acridum
Source: Front Microbiol. 2020 May 5;11:849. doi: 10.3389/fmicb.2020.00849 (PMC7214794; doi:10.3389/fmicb.2020.00849)
Supplement: Supplementary file 1 [file Data_Sheet_1.PDF]

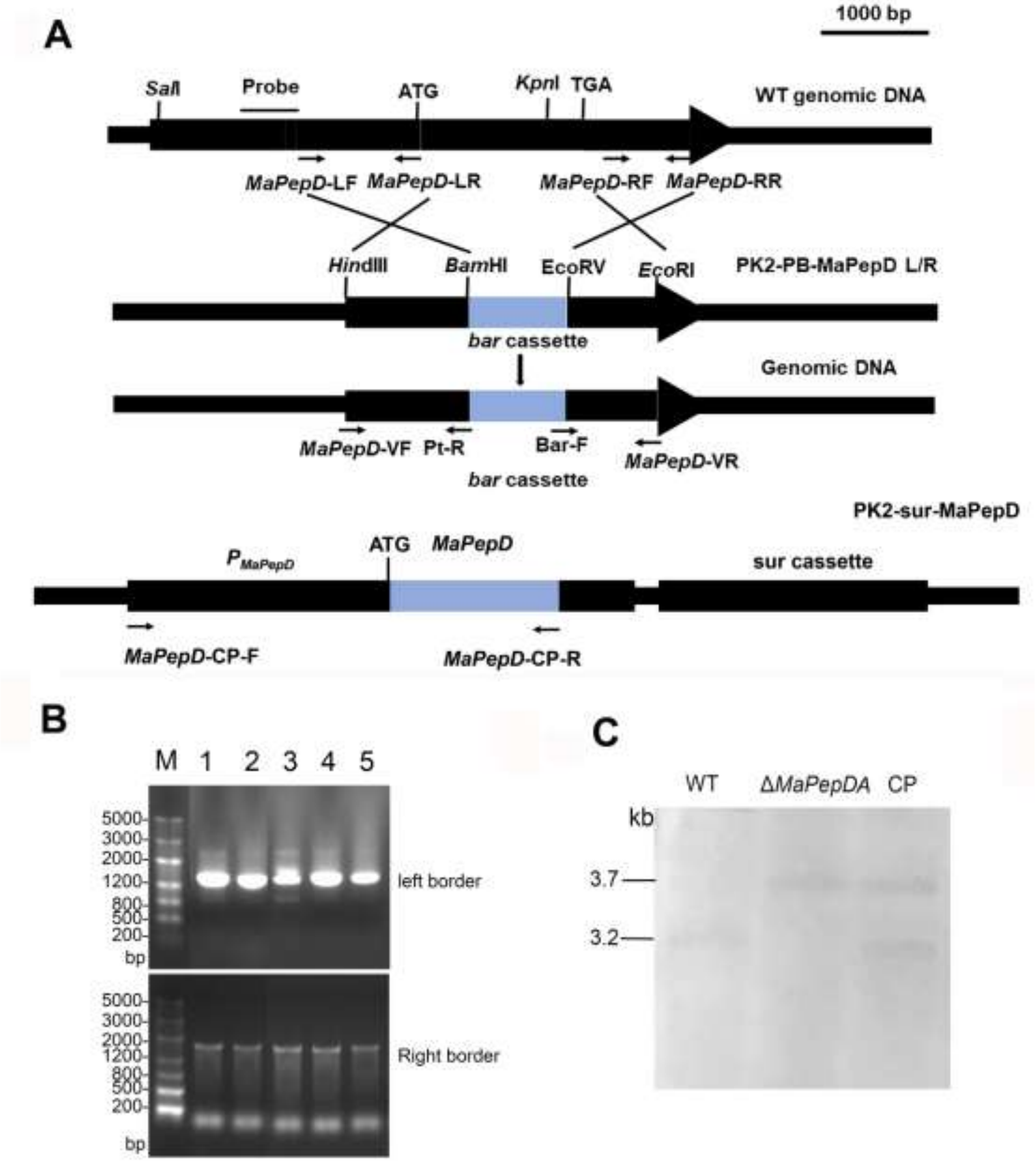

FIGURE S1 Disruption and complementation of *MaPepDA* in *M. acridum*. (A) The *MaPepDA* was disrupted by homologous recombination method. The plasmid pK2-PB-MaPepDA contains a Bar cassette. Complementing vector of *MaPepDA* carried a *MaPepDA* gene and upstream regulation sequence. (B) Verification of mutant by PCR. (C) Verification of mutant by Southern blotting. The genomic DNAs were cut with *Sal*I and *Kpn*I. The probe was amplified from genomic DNAs by PCR using the primers in Table S1.

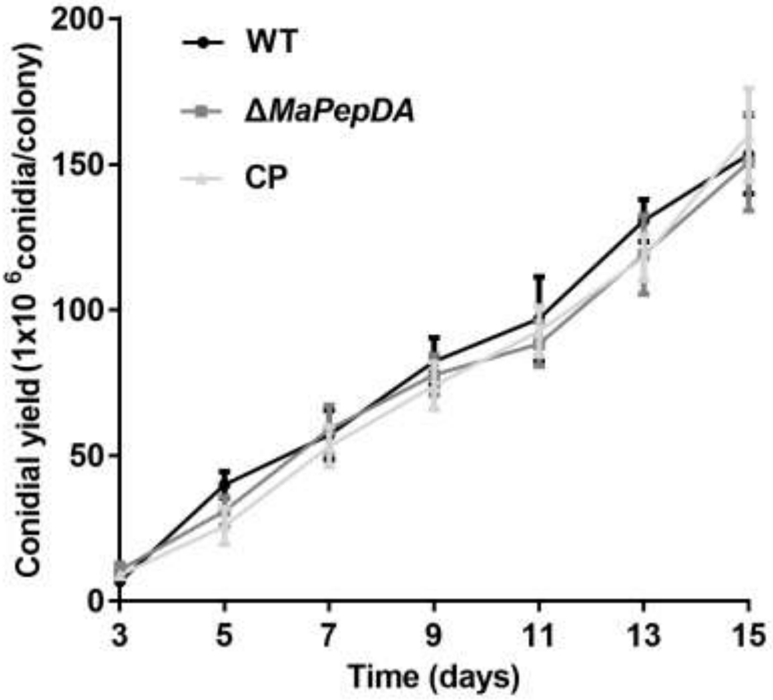

FIGURE S2 Time-course analysis of conidial yield. Aliquots of 5 μl of 1×10^7 conidia/ml of fungal strains were spotted on 1/4 SDAY. The colony were collected and suspended in 1 ml Tween 80, and then vortexed for 10 min. The suspensions were filtered with lens tissue. The concentration of conidia suspension was determined with a hemocytometer under microscope.

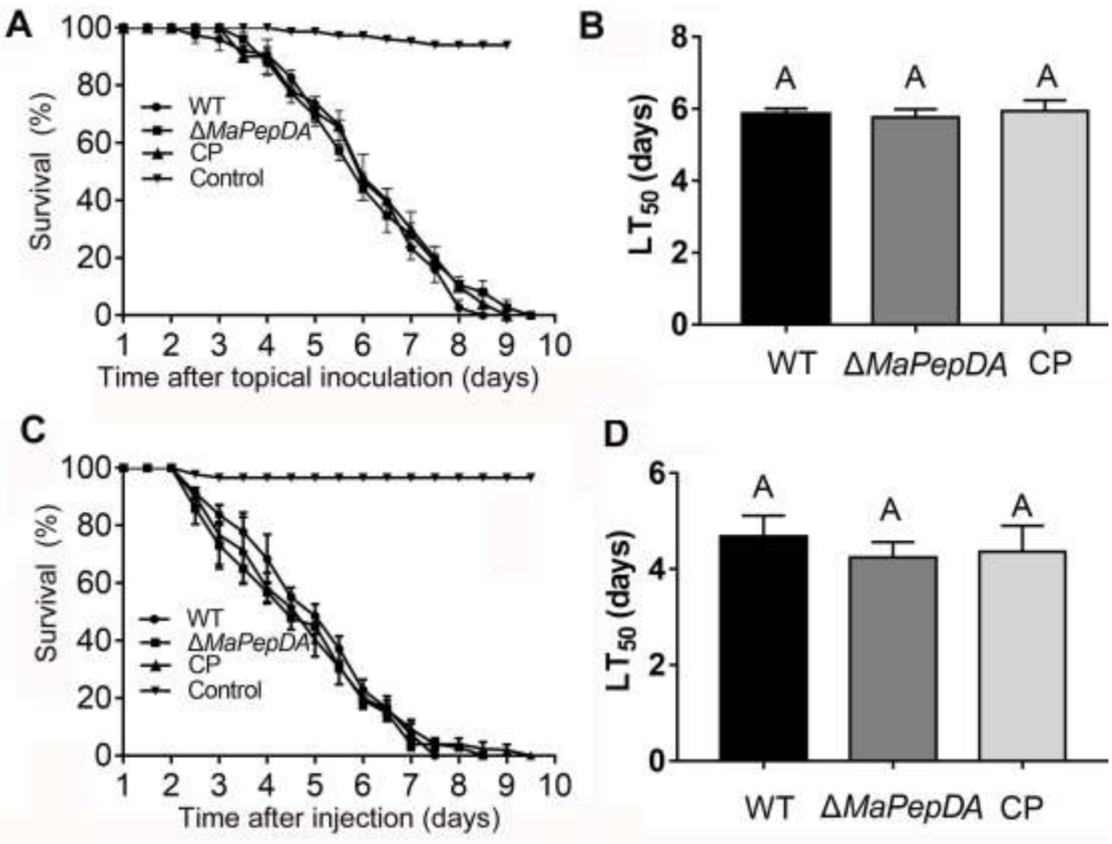

FIGURE S3 Insect bioassays. (A) Insect survival after topical application of conidia of WT, Δ*MaPepDA*, or complemented strain. Liquid paraffin oil was the blank control. (B) LT_{50} for topical inoculation assay. (C) Insect survival after injection of conidia directly into the insect hemocoel. Sterile water was the blank control. (D) LT_{50} for injection assay. Error bars are standard deviations of three trials. Same letters above the columns indicate no significant difference.

TABLE S1 Primers used in this study

Primer	Sequence (5′-3′)*	Remarks
cDNA-F	ATGAAGCCTCATCTCGCGGCC	Clone the cDNA of
cDNA-R	TCAAGACAGATCCTGGACAACA	*MaPepDA*
MaPepD-LF	GACGGCCAGTGCCAAGCT TACCACCCAGTCGGAGCA	Disruption transformants
MaPepD-LR	CGGATCCCTCGAGTCTAG AGGGTCTGCGGAACAAGC	
MaPepD-RF	GCTGGCCGCCCATGGGATAAGCGACGACAGCAAAGACA	Disruption transformants
MaPepD-RR	ATGACATGATTACGAATTCGAAGCATACGGAGCAGAAG	
MaPepD-VF	GACGAGTGAGATTAGGGCTTTC	Screening the *MaPepDA*
LB-PT-R	CAGCCAAGCCCAAAAAGTG	
RB-Bar-F	GCTCTACACCCACCTGCT	Screening the *MaPepDA*
MaPepD-VR	GCCCCTGTAATGAAGACGAG	
MaPepD-PF	TTTGTTCCCATATTACGCAGTT	Clone the probe
MaPepD-PR	AAGCCCTAATCTCACTCGTCT	
MaPepD-CP-F	GACGGCCAGTGCCAAGCTCGACCGAGCTTCTCAACAGGG	Clone complementation
MaPepD-CP-R	CCTTGCTCACCATGGATCCAGACAGATCCTGGACAACAA	of *MaPepDA*
MaPepD-CP-VF	CTACGGCGGAAACACGCACCT	Verify the vector of CP
EGFP-VR	CGATGCGGTTCACCAGGGTGT	
EX-F	ATTTCCCCTAAGTACTTCTAGAATGAAGCCTCATCTC	Clone the overexpression
EX-R	TTGCTCACCATGGATCCAGACAGATCCTGGACAACAA	
Eo-F	TTAAGAAGGAGATATACCATGAAGCCTCATCTCGCGG	Clone the cDNA of prokaryotic expression in *E. coil*
Eo-R	GTGGTGGTGGTGGTGGTGCTCGAGAGACAGATCCTGGACAAC	

TABLE S2 Differential expressed genes between WT and Δ*MaPepDA* strains

Gene ID	Product name	Log_2^{ratio} (Δ*MaPePDA*/WT)	References
**Amino acid metabolism**			
MAC_06861	Aldehyde dehydrogenase	2.42917446	(Asiimwe et al., 2012)
MAC_08739	Carboxypeptidase	1.48806815	(St Leger et al., 1994)
MAC_04079	Amino-acid permease inda1	1.15615597	(Sauer, 1984)
MAC_02956	Tyrosinase	1.14412774	(Yang et al., 2011)
MAC_04483	Proline oxidase Put1, putative	1.12026531	(Wang and Brandriss, 1986)
MAC_08901	Aspartate aminotransferase, putative	1.42247981	(Wang et al., 2016)
MAC_06811	Alkaline serine protease AorO, putative	1.08846785	(Hui et al., 2004)
MAC_07959	Hydantoinase/oxoprolinase, putative	1.01141874	(Ye et al., 1996)
MAC_06980	Peptide synthetase 3	-1.18111933	(McErlean et al., 2019)
MAC_08095	Amidohydrolase family protein	1.42917446	(Sadowsky et al., 1998)
**DNA replication and repair**			
MAC_07294	Cysteine rich protein	1.69000796	(Szczypka and Thiele, 1989)
MAC_07387	4-coumarate-CoA ligase 2	1.37364152	(Kuhn et al., 1984)
MAC_01848	Indole-diterpene biosynthesis protein PaxU, putative	1.16614006	(Young et al., 2005)
MAC_09373	A/G-specific adenine glycosylase	1.008382995	(Eberle et al., 2015)
**Growth and development**			
MAC_06859	MFS multidrug transporter, putative	1.199079001	(Crespo-Sempere et al., 2014)
MAC_05794	3,2-trans-enoyl-CoA isomerase precursor, putative	1.050895513	(Gurvitz et al., 1998)
MAC_07845	Cytochrome P450	1.10473951	(Shin et al., 2017)
MAC_05691	Major facilitator superfamily domain containing protein 5	1.64216819	(Berger et al., 2012)
MAC_08428	NDT80 / PhoG like DNA-binding family protein	-2.22687114	(Doyle et al., 2016)
MAC_07120	Cytochrome P450 52A11	1.38502736	(Breskvar et al., 1995)
MAC_07320	Sugar transporter, putative	-2.187496898	(Zhang et al., 2011)
MAC_01730	Sugar transporter, putative	1.16902257	(Zhang et al., 2011)
MAC_00175	Glucose dehydrogenase, putative	1.26502096	(Maleki et al., 2015)
MAC_08696	Endo-N-acetyl-beta-D-glucosaminidase precursor	1.45259881	(Pusztahelyi and Pócsi, 2014)
MAC_06293	Type I phosphodiesterase/nucleotide pyrophosphatase	-1.05944417	(Yang et al., 2017)
MAC_06812	C-3 sterol dehydrogenase/C-4 decarboxylase	1.277171369	(Aaron et al., 2001)
MAC_02181	Monooxygenase, putative	1.0769198	(Deng et al., 2018)
MAC_00080	C2H2 finger domain protein, putative	1.24360822	(Huang et al., 2005)
MAC_00167	Extracellular dioxygenase, putative	1.19700102	(Boissel et al., 2009)
MAC_06584	Glucose repressible protein Grg1	1.31893306	(Lee and Moss, 1993)
MAC_06154	Transcriptional regulatory protein pro-1	1.29531872	(Ryder et al., 2004)
MAC_04470	Catalase	-1.148102901	(Hansberg et al., 2012)
MAC_05139	C6 zinc finger domain protein	1.04299183	(Masloff et al., 2002)
MAC_09507	Hydrophobin	-1.49487668	(Wessels et al., 1991)
**Sporulation**			

MAC_08744	SpoC1-C1C	2.59909946	(Stephens et al., 1999)
MAC_08903	MFS transporter, putative	1.0006327	(Khaokhajorn et al., 2015)
MAC_02934	Glucose-methanol-choline oxidoreductase	-1.30595991	(Etxebeste et al., 2012)
**Cell wall components**			
MAC_06615	Putative cyclic nucleotide gated channel beta 1	1.5166373	(Thomas et al., 2001)
MAC_05133	Cell wall protein	1.3514112	(Kirkham et al., 2017)
MAC_02205	Chitinase 18-15	1.32533865	(Katarina et al., 2010)
MAC_09698	Oxidoreductase, 2OG-Fe (II) oxygenase family	1.18014692	(Fang et al., 2012)
MAC_02204	Cell wall protein	1.4692519	(Ouyang et al., 2019)
MAC_04535	Inositol monophosphatase	1.87211796	(Goswami et al., 2018)
MAC_08097	Putative chitosanase CSN1	1.03220854	(Liu et al., 2010)
MAC_06312	Gram-positive signal peptide, ysirk family	1.48992252	(DeDent et al., 2008)
MAC_08916	Cell surface protein	-1.64882805	(Wong et al., 2007)
MAC_05852	Antigenic cell wall galactomanno protein	1.26129729	(Chan et al., 2002)
MAC_05145	Glycosyl hydrolase, family 18, putative	-1.14709034	(Wu et al., 2001)
**Stress resistance**			
MAC_01566	MFS monosaccharide transporter, putative	1.35528704	(Lopez-Moya et al., 2016)
MAC_08131	RING-1 like protein	1.23510731	(Daumke et al., 2010)
MAC_06860	Nonribosomal peptide synthase, putative	1.17919621	(Oide et al., 2006)
MAC_05717	ThiJ/PfpI domain-containing protein	1.16770735	(Bankapalli et al., 2015)
MAC_01513	RTA1 domain protein, putative	-1.51455732	(Mulcahey et al., 2009）
MAC_01732	Benzoate 4-monooxygenase	1.12105217	(Shinji et al., 1998)
MAC_04555	Benzoate 4-monooxygenase cytochrome P450, putative	2.1644095	(Shinji et al., 1998)
MAC_05903	Cysteine desulfurase	1.00531311	(Sandrine et al., 2003)
MAC_08307	Myo-inositol oxygenase	1.08355836	(Zhan et al., 2015)
MAC_09501	MFS quinate transporter, putative	-6.365860188	(Hoffmann et al. 2003)
MAC_09183	Dihydrofolate reductase	-1.49776208	(Gorelova et al., 2017)
MAC_04778	Nicotinamide riboside kinase	2.12961418	(Belenky et al., 2009)
**Carbohydrate metabolism**			
MAC_06644	(S)-2-hydroxy-acid oxidase, putative	1.16941519	(Zhang et al., 2019)
MAC_05415	2,6-dihydropseudooxynicotine hydrolase	-3.53335083	(Sachelaru et al.,2005)
MAC_02142	Endo alpha-1,4 polygalactosaminidase precusor	1.0416177	(Naumov and tepushchenko, 2011)
MAC_00989	Sugar transporter (hexose transporter)	1.1963403	(Ozcan and Johnston, 1995)
MAC_07957	Lactate dehydrogenase	1.31791771	(Gleason and Nolan, 1966)
**Energy metabolism**			
MAC_07816	Inorganic pyrophosphatase	-1.10428353	(de Meis, 1985)
MAC_03493	Nitrite reductase	-1.20801882	(Wijma et al., 2007)
MAC_08624	Nitrate reductase	-1.72398696	(Wijma et al., 2007)
MAC_08430	Oxidoreductase, 2-nitropropane dioxygenase family	1.00681628	(Kido and Soda, 1978)
MAC_08721	Flavin-binding monooxygenase	1.18361288	(Xiao et al., 2012)
MAC_00200	Pfs, NACHT and WD domain protein	1.00119791	(Espagne et al., 1997)

**Lipid metabolism**			
MAC_09442	Putative long-chain-fatty-acid--CoA ligase FAA2	1.19668707	(Murphy and Spence, 1980)
MAC_05853	Hydrophobic surface binding protein	1.734451317	(Saharine and Keski-Oja, 2000)
MAC_01452	Pfs, NACHT and Ankyrin domain protein	1.09075825	(Zhou et al., 2000)
MAC_09815	Fatty acid-binding protein FABP, putative	1.01413696	(Niot et al., 1997)
MAC_06816	Putative cholesterol oxidase precursor	1.10082508	(Ghosh et al., 2018)
MAC_00274	Enoyl-CoA hydratase/carnithine racemase	1.10528485	(Liu et al., 2016)
MAC_08994	Esterase/lipase	-1.66704085	(Valek et al., 2019)
MAC_08900	phytanoyl-CoA dioxygenase family protein	1.13343589	(Masuda et al., 1967)
MAC_06273	Arrestin domain-containing protein	1.01047065	(Ogawa et al., 2019)
MAC_09347	3-ketoacyl-acyl carrier protein reductase, putative	1.16614006	(Cheng et al., 2012)
**Virulence**			
MAC_06641	Allergen	2.1644095	(Coleman et al., 1975)
MAC_03516	Toxin biosynthesis protein, putative	1.19793414	(Aranda et al., 2018)
**Hypothetical protein**			
MAC_08031	Hypothetical protein	1.05895547	
MAC_08454	Hypothetical protein	1.43699397	
MAC_04777	Hypothetical protein	1.36206027	
MAC_02043	Hypothetical protein	-1.30875906	
MAC_05633	Hypothetical protein	1.09008582	
MAC_09701	Hypothetical protein	1.11174776	
MAC_07196	Hypothetical protein	-1.59314012	
MAC_06795	Hypothetical protein	2.59909946	
MAC_08697	Hypothetical protein	1.70340213	
MAC_08698	Hypothetical protein	1.47569712	
MAC_09032	hypothetical protein	1.36777392	
MAC_01405	Hypothetical protein	1.34171162	
MAC_05692	Hypothetical protein	1.33147779	
MAC_07530	Hypothetical protein	1.31129715	
MAC_07870	Hypothetical protein	1.28952247	
MAC_08745	Hypothetical protein	1.2809235	
MAC_04545	Hypothetical protein	1.21117381	
MAC_02851	Hypothetical protein	1.25950272	
MAC_09514	Hypothetical protein	1.25514506	
MAC_02850	Hypothetical protein	1.25067357	
MAC_09149	Hypothetical protein	1.24762709	
MAC_09700	Hypothetical protein	1.22644057	
MAC_06079	Hypothetical protein	1.21676613	
MAC_03322	Hypothetical protein	1.17389578	
MAC_00321	Hypothetical protein	1.12734757	
MAC_02603	Hypothetical protein	1.112765	
MAC_08016	Hypothetical protein	1.099908	
MAC_06965	Hypothetical protein	1.08843753	

MAC_08010	Hypothetical protein	1.07726494	
MAC_01234	Hypothetical protein	1.07687272	
MAC_02044	Hypothetical protein	1.04110401	
MAC_09469	Hypothetical protein	1.02830878	
MAC_06538	Hypothetical protein	1.01413696	
MAC_00261	Hypothetical protein	1.01413696	
MAC_04559	Hypothetical protein	-3.79321796	
MAC_06978	Hypothetical protein	-3.30779113	
MAC_05095	Hypothetical protein	-2.86447664	
MAC_05396	Hypothetical protein	-2.83830585	
MAC_03274	Hypothetical protein	-1.56255444	
MAC_09326	Hypothetical protein	-1.41235916	
MAC_01218	Hypothetical protein	-1.40967075	
MAC_08896	Hypothetical protein	-1.40090054	
MAC_05392	Hypothetical protein	-1.32466495	
MAC_02230	Hypothetical protein	-1.2374018	
MAC_02290	Hypothetical protein	-1.20045709	
MAC_04973	Hypothetical protein	-1.08562447	
MAC_01193	Hypothetical protein	-2.148224744	
MAC_06307	Hypothetical protein	-1.03255627	
MAC_09561	Hypothetical protein	-1.01782895	

References

Aaron, K.E., Pierson, C. A., Lees, N.D., and Bard, M. (2001). The *Candida albicans* ERG26 gene encoding the C-3 sterol dehydrogenase (C-4 decarboxylase) is essential for growth. FEMS. Yeast. Res. 1(2):93-101.

Aranda, J.F., Rathjen S., Johannes, L., Fernández-Hernando, C. (2018). MicroRNA 199a-5p Attenuates Retrograde Transport and Protects against Toxin-Induced Inhibition of Protein Biosynthesis. Mol. Cell. Biol. 38(11). pii: e00548-17.

Asiimwe, T., Krause, K., Schlunk, I., and Kothe, E. (2012). Modulation of ethanol stress tolerance by aldehyde dehydrogenase in the mycorrhizal fungus *Tricholoma vaccinum*. Mycorrhiza. 22(6):471-84.

Bankapalli, K., Saladi, S., Awadia, S. S, Goswami, A.V., Samaddar, M., and D'Silva, P. (2015). Robust glyoxalase activity of Hsp31, a ThiJ/DJ-1/PfpI family member protein, is critical for oxidative stress resistance in *Saccharomyces cerevisiae*. J. Biol. Chem. 290(44):26491-507.

Belenky, P., Christensen, K.C., Gazzaniga, F., Pletnev, A. A., and Brenner, C. (2009). Nicotinamide riboside and nicotinic acid riboside salvage in fungi and mammals. Quantitative basis for Urh1 and purine nucleoside phosphorylase function in NAD+ metabolism. J. Biol. Chem. 284(1):158-64.

Berger, J.H., Charron, M.J., and Silver, D.L. (2012). Major facilitator superfamily domain-containing protein 2a (MFSD2A) has roles in body growth, motor function, and lipid metabolism. PLoS. One. 7(11): e50629.

Boissel, S., Reish, O., Proulx, K., Kawagoe-Takaki, H., Sedgwick, B., Yeo, G.S., Meyre, D., Golzio, C., Molinari, F., Kadhom, N., Etchevers, H.C., Saudek, V., Farooqi, I.S., Froguel, P.,

Lindahl, T., O'Rahilly, S., Munnich, A., and Colleaux, L. (2009). Loss-of-function mutation in the dioxygenase-encoding FTO gene causes severe growth retardation and multiple malformations. Am. J. Hum. Genetv. 85(1):106-11.

Breskvar, K., Ferencak, Z., and Hudnik-Plevnik, T. (1995). The role of cytochrome P450(11 alpha) in detoxification of steroids in the filamentous fungus *Rhizopus nigricans*. J. Steroid. Biochem. Mol. Biol. 52(3):271-5.

Chan, C. M., Woo, P. C., Leung, A. S., Lau, S. K., Che, X. Y., Cao, L., and Yuen, K. Y. (2002). Detection of antibodies specific to an antigenic cell wall galactomannoprotein for serodiagnosis of Aspergillus fumigatus aspergillosis. J. Clin. Microbiol. 40(6):2041-5.

Cheng, J., Ma, J., Lin, J., Fan, Z. C., Cronan, J.E., and Wang, H. (2012). Only one of the five *Ralstonia solanacearum* long-chain 3-ketoacyl-acyl carrier protein synthase homologues functions in fatty acid synthesis. Appl. Environ. Microbiol. 78(5):1563-73.

Coleman, G., Brown, S., and Stormonth, D. A. (1975). A model for the regulation of bacterial extracellular enzyme and toxin biosynthesis. J. Theor. Biol. 52(1):143-8.

Crespo-Sempere, A., Martínez-Culebras, P.V., González-Candelas, L. (2014). The loss of the inducible *Aspergillus carbonarius* MFS transporter MfsA leads to ochratoxin A overproduction. Int. J. Food. Microbiol. 181:1-9.

Daumke, O., Gao, S., von der, M. A., Haller, O., and Kochs, G. (2010). Structure of the MxA stalk elucidates the assembly of ring-like units of an antiviral module. Small GTPases 1(1):62-64

de Meis, L. (1985). Role of water in processes of energy transduction: Ca2+-transport ATPase and inorganic pyrophosphatase. Biochem. Soc. Symp. 50:97-125.

DeDent, A., Bae, T., Missiakas, D. M., and Schneewind, O. (2008). Signal peptides direct surface proteins to two distinct envelope locations of *Staphylococcus aureus*. EMBO. J. 27(20):2656-68.

Deng, H., Gao, R., Liao, X., and Cai, Y. (2018). Characterisation of a monooxygenase in *Shiraia bambusicola*. Microbiology. 164(9):1180-1188.

Doyle, C. E., Kitty, Cheung, H.Y., Spence, K.L., and Saville, B.J. (2016). Unh1, an Ustilago maydis Ndt80-like protein, controls completion of tumor maturation, teliospore development, and meiosis. Fungal. Genet. Biol. 94:54-68.

Eberle, R. J., Coronado, M. A., Caruso, I. P., Lopes, D. O., Miyoshi, A., Azevedo, V., and Arni, R. K. (2015). Chemical and thermal influence of the [4Fe-4S]2+ cluster of A/G-specific adenine glycosylase from *Corynebacterium pseudotuberculosis*. Biochim. Biophys. Acta. 1850(2):393-400.

Eberle, R. J., Coronado, M. A., Caruso, I. P., Lopes, D. O., Miyoshi, A., Azevedo, V., Arni, R. K. (2015). Chemical and thermal influence of the [4Fe-4S]2+ cluster of A/G-specific adenine glycosylase from *Corynebacterium pseudotuberculosis*. Biochim. Biophys. Acta. 1850(2):393-400.

Espagne, E., Balhadère, P., Bégueret, J., and Turcq, B. (1997). Reactivity in vegetative incompatibility of the HET-E protein of the fungus *Podospora anserina* is dependent on GTP-binding activity and a WD40 repeated domain. Mol. Gen. Genet. 256(6):620-7.

Etxebeste, O., Herrero-García, E., Cortese, M. S., Garzia, A., Oiartzabal-Arano, E., de los Ríos, V., Ugalde, U., and Espeso, E.A. (2012). GmcA is a putative glucose-methanol-choline oxidoreductase required for the induction of asexual development in *Aspergillus nidulans.* PLoS. One. 7(7): e40292.

Fang, L., Zhao, F., Cong, Y., Sang, X., Du, Q., Wang, D., Li, Y., Ling, Y., Yang, Z., and He, G. (2012). Rolling-leaf14 is a 2OG-Fe (II) oxygenase family protein that modulates rice leaf rolling by affecting secondary cell wall formation in leaves. Plant. Biotechnol. J. 10(5):524-32.

Ghosh, S., Ahmad, R., Gautam, V. K., and Khare, S. K. (2018). Cholesterol-oxidase-magnetic nanobioconjugates for the production of 4-cholesten-3-one and 4-cholesten-3, 7-dione. Bioresour. Technol. 254:91-96.

Gleason, F. H., and Nolan, R. A. (1966). D (-)-lactate dehydrogenase in lower fungi. Science 152(3726):1272-3.

Gorelova, V., De Lepeleire, J., Van Daele, J., Pluim, D., Meï, C., Cuypers, A., Leroux, O., Rébeillé, F., Schellens, J. H. M., Blancquaert, D., Stove, C.P., and Van Der Straeten, D. (2017). Dihydrofolate reductase/thymidylate synthase fine-tunes the folate status and controls redox homeostasis in plants. Plant. Cell. 29(11):2831-2853.

Goswami, R., Bondoc, J.M.G., Wheeler, P.R., Jafari, A., Gonzalez, T., Mehboob, S., and Movahedzadeh, F. (2018). Inositol Monophosphatase: A Bifunctional Enzyme in *Mycobacterium smegmatis*. ACS. Omega. 3(10):13876-13881.

Gurvitz, A., Mursula, A. M., Firzinger, A., Hamilton, B., Kilpeläinen, S. H., Hartig, A., Ruis, H., Hiltunen, J. K., Rottensteiner, H. (1998). Peroxisomal Delta3-cis-Delta2-trans-enoyl-CoA isomerase encoded by ECI1 is required for growth of the yeast *Saccharomyces cerevisiae* on unsaturated fatty acids. J. Biol. Chem. 273(47):31366-74.

Gurvitz, A., Mursula, A.M., Firzinger, A., Hamilton, B., Kilpeläinen, S.H., Hartig, A., Ruis, H., Hiltunen, J.K., and Rottensteiner, H. (1998). Peroxisomal Delta3-cis-Delta2-trans-enoyl-CoA isomerase encoded by ECI1 is required for growth of the yeast *Saccharomyces cerevisiae* on unsaturated fatty acids. J. Biol. Chem. 273(47):31366-74.

Hansberg, W., Salas-Lizana, R., and Domínguez, L. (2012). Fungal catalases: function, phylogenetic origin and structure. Arch. Biochem. Biophys. 525(2):170-80.

Hoffmann, L., Maury, S., Martz, F., Geoffroy, P., Legrand, M. (2003). Purification, cloning, and properties of an acyltransferase controlling shikimate and quinate ester intermediates in phenylpropanoid metabolism. J. Biol. Chem. 278(1):95-103.

Huang, J., Wang, J., and Zhang, H. (2005). Rice ZFP15 gene encoding for a novel C2H2-type zinc finger protein lacking DLN box, is regulated by spike development but not by abiotic stresses. Mol. Biol. Rep. 32(3):177-83.

Hui, Z., Doi, H., Kanouchi, H., Matsuura, Y., Mohri, S., Nonomura, Y., and Oka, T. (2004). Alkaline serine protease produced by *Streptomyces* sp. degrades PrP (Sc). Biochem. Biophys. Res. Commun. 321(1):45-50.

Katarina, I., Nashwan, A., Wimal, U., Petter, M., Jan, S., and Magnus, K. (2010). Comparative molecular evolution of *Trichoderma Chitinases* in response to mycoparasitic interactions. Evol. Bioinform. Online. 6: 1-26.

Khaokhajorn, P., Samipak, S., Nithithanasilp, S., Tanticharoen, M., and Amnuaykanjanasin, A. (2015). Production and secretion of naphthoquinones is mediated by the MFS transporter MFS1 in the entomopathogenic fungus *Ophiocordyceps* sp. BCC1869. World. J. Microbiol. Biotechnol. 31(10):1543-54.

Kido, T., and Soda, K. (1978). Properties of 2-nitropropane dioxygenase of *Hansenula mrakii*. Formation and participation of superoxide. J. Biol. Chem. 253(1):226-32.

Kirkham, A. R., Richthammer, P., Schmidt, K., Wustmann, M., Maeda, Y., Hedrich, R., Brunner, E., Tanaka, T., van Pée, K. H., Falciatore, A., and Mock, T. (2017). A role for the cell-wall protein silacidin in cell size of the diatom *Thalassiosira pseudonana*. ISME. J. 11(11):2452-2464.

Kuhn, D. N., Chappell, J., Boudet, A., and Hahlbrock, K. (1984). Induction of phenylalanine ammonia-lyase and 4-coumarate: CoA ligase mRNAs in cultured plant cells by UV light or fungal elicitor. Proc. Natl. Acad. Sci. U S A. 81(4):1102-6.

Lee, F. J., and Moss, J. (1993). An RNA-binding protein gene (RBP1) of *Saccharomyces cerevisiae* encodes a putative glucose-repressible protein containing two RNA recognition motifs. J. Biol. Chem. 268(20):15080-7.

Liu, G., Cai, S., Hou, J., Zhao, D., Han, J., Zhou, J., and Xiang, H. (2016). Enoyl-CoA hydratase mediates polyhydroxyalkanoate mobilization in *Haloferax mediterranei*. Sci. Rep. 6:24015.

Liu, H., Zhang, B., Li, C., and Bao, X. (2010). Knock down of chitosanase expression in phytopathogenic fungus *Fusarium solani* and its effect on pathogenicity. Curr. Genet. 56(3):275-81.

Lopez-Moya, F., Kowbel, D., Nueda, M. J., Palma-Guerrero, J., Glass, N. L., and Lopez-Llorca, L. V. (2016). *Neurospora crass*a transcriptomics reveals oxidative stress and plasma membrane homeostasis biology genes as key targets in response to chitosan. Mol. Biosyst. 12(2):391-403.

Maleki, S., Mærk, M., Valla, S., and Ertesvåg, H. (2015). Mutational analyses of glucose dehydrogenase and glucose-6-phosphate dehydrogenase genes in *Pseudomonas fluorescens* reveal their effects on growth and alginate production. Appl. Environ. Microbiol. 81(10):3349-56.

Masloff, S., Jacobsen, S., Pöggeler, S., and Kück, U. (2002). Functional analysis of the C6 zinc finger gene pro1 involved in fungal sexual development. Fungal. Genet. Biol. 36(2):107-16.

Masuda, Y., Mori, K., and Kuratsune, M. (1967). Polycyclic aromatic hydrocarbons formed by pyrolysis of carbohydrates, amino acids, and fatty acids. Gan. 58(1):69-74.

McErlean, M., Overbay, J., and Van Lanen, S. (2019). Refining and expanding nonribosomal peptide synthetase function and mechanism. J. Ind. Microbiol. Biotechnol. 6(3-4):493-513.

Mulcahey, M., Thakur, N., Tocci, S., and Eberson, C. (2009). Compartment syndrome in a child secondary to acute osteomyelitis of the ulna. Pediatr. Infect. Dis. J. 28(3):258-9.

Murphy, M.G., and Spence, M.W. (1980). Long-chain fatty acid:CoA ligase in rat brain in vitro: a comparison of activities with oleic and cis-vaccenic acids. J. Neurochem. 34(2):367-73.

Naumov, D. G, and Stepushchenko, O. O. (2011). Endo-alpha-1-4-polygalactosaminidases and their homologues: structure and evolution. Mol. Biol. 45(4):703-14.

Niot, I., Poirier, H., and Besnard, P. (1997). Regulation of gene expression by fatty acids: special reference to fatty acid-binding protein (FABP). Biochimie. 79(2-3):129-33.

Ogawa, M., Kanda, T., Higuchi, T., Takahashi, H., Kaneko, T., Matsumoto, N., Nirei, K., Yamagami, H., Matsuoka, S., Kuroda, K., and Moriyama, M. (2019). Possible association of arrestin domain-containing protein 3 and progression of non-alcoholic fatty liver disease. Int J. Med. Sci. 16(7):909-921.

Oide, S., Moeder, W., Krasnoff, S., Gibson, D., Haas, H., Yoshioka, K., and Turgeon, B. G. (2006). NPS6, encoding a nonribosomal peptide synthetase involved in siderophore-mediated iron metabolism, is a conserved virulence determinant of plant pathogenic ascomycetes.

Plant. Cell. 18(10):2836-53.

Ouyang, X., Dong, C. L., and Ubogu, E. E. (2019). In situ molecular characterization of endoneurial microvessels that form the blood-nerve barrier in normal human adult peripheral nerves. J. Peripher. Nerv. Syst. 24(2).

Ozcan, S., and Johnston, M. (1995). Three different regulatory mechanisms enable yeast hexose transporter (HXT) genes to be induced by different levels of glucose. Mol. Cell. Biol. 15(3):1564-72.

Pusztahelyi, T., and Pócsi, I. (2014). Chitinase but N-acetyl-β-D-glucosaminidase production correlates to the biomass decline in Penicillium and *Aspergillus species*. Acta. Microbiol. Immunol. Hung. 61(2):131-43.

Ryder, S. P., Frater, L. A., Abramovitz, D. L., Goodwin, E. B., and Williamson, J. R. (2004). RNA target specificity of the STAR/GSG domain post-transcriptional regulatory protein GLD-1. Nat. Struct. Mol. Biol. 11(1):20-8.

Sachelaru, P., Schiltz, E., Igloi, G. L., and Brandsch, R. (2005). An alpha/beta-fold C-C bond hydrolase is involved in a central step of nicotine catabolism by *Arthrobacter nicotinovorans*. J. Bacteriol. 187(24):8516-9.

Sadowsky, M.J., Tong, Z., de Souza, M., and Wackett, L. P. (1998). AtzC is a new member of the amidohydrolase protein superfamily and is homologous to other atrazine-metabolizing enzymes. J. Bacteriol. 180(1):152-8.

Saharine, J., and Keski-Oja, J. (2000). Specific sequence motif of 8-Cys repeats of TGF-beta binding proteins, LTBPs, creates a hydrophobic interaction surface for binding of small latent TGF-beta. Mol. Biol. Cell. 11(8):2691-704.

Sandrine, O. C., Lascoux, D., Loiseau, L., Barras, F., Forest, E., and Fontecave, M. (2003). Mechanistic studies of the SufS-SufE cysteine desulfurase: evidence for sulfur transfer from SufS to SufE. FEBS. Lett. 555(2):263-7.

Sauer, N. (1984). A general amino-acid permease is inducible in Chlorella vulgaris. Planta. 161(5):425-31.

Shin, J. Y., Bui, D. C., Lee, Y., Nam, H., Jung, S., Fang, M., Kim, J. C., Lee, T., Kim, H., Choi, G. J., Son, H., and Lee, Y. W. (2017). Functional characterization of cytochrome P450 monooxygenases in the cereal head blight fungus *Fusarium graminearum*. Enviro. Microbiol. 2017, 19(5):2053-2067.

Shinji, K., Weerashinghe, I.S., and Toshio, S. (1998). P450 monooxygenases are an important mechanism of permethrin resistance in *Culex quinquefasciatus* Say larvae. Arch. Insect. Biochem. 1 (1998) 37:13.

St Leger, R. J., Bidochka, M. J., and Roberts, D.W. (1994). Characterization of a novel carboxypeptidase produced by the entomopathogenic fungus *Metarhizium anisopliae*. Arch. Biochem. Biophys. 314(2):392-8.

Stephens, K. E., Miller, K. Y., and Miller, B. L. (1999). Functional analysis of DNA sequences required for conidium-specific expression of the SpoC1-C1C gene of *Aspergillus nidulans*. Fungal. Genet. Biol. 27(2-3):231-42.

Szczypka, M. S., and Thiele, D. J. (1989). A cysteine-rich nuclear protein activates yeast metallothionein gene transcription. Mol. Cell. Biol. 9(2):421-9.

Thomas, C.R., Tonia, E. T., Rohan, J. G., Schaack, J., and Karpena, J. W. (2001). In vivo assessment of local phosphodiesterase activity using tailored cyclic nucleotide-gated

channels as camp sensors. J. Gen. Physiol. 118(1): 63-78.

Valek, T., Kostelnik, A., Valkova, P., and Pohanka, M. (2019). Indoxyl Acetate as a Substrate for Analysis of Lipase Activity. Int. J. Anal. Chem. 2019:8538340.

Wang, R., Zhang, M., Liu, H., Xu, J., Yu, J., He, F., Zhang, X., Dong, S., and Dou, D. (2016). PsAAT3, an oomycete-specific aspartate aminotransferase, is required for full pathogenicity of the oomycete pathogen *Phytophthora sojae*. Fungal. Biol. 120(4):620-630.

Wang, S. S., and Brandriss, M. C. (1986). Proline utilization in *Saccharomyces cerevisiae*: analysis of the cloned PUT1 gene. Mol. Cell. Biol. 6(7): 2638-2645.

Wessels, J., De Vries, O., Asgeirsdottir, S. A., and Schuren, F. (1991). Hydrophobin genes involved in formation of aerial hyphae and fruit bodies in *Schizophyllum*. Plant. Cell. 3(8):793-799.

Wijma, H.J., MacPherson, I., Farver, O., Tocheva, E.I., Pecht, I., Verbeet, M. P., Murphy, M. E., and Canters, G. W. (2007). Effect of the methionine ligand on the reorganization energy of the type-1 copper site of nitrite reductase. J. Am. Chem. Soc. 129(3):519-25.

Wong Sak Hoi, J., Herbert, C., Bacha, N., O'Connell, R., Lafitte, C., Borderies, G., Rossignol, M., Rougé, P., and Dumas, B. (2007). Regulation and role of a STE12-like transcription factor from the plant pathogen *Colletotrichum lindemuthianum*. Mol. Microbiol. 64(1):68-82.

Wu, Y., Egerton, G., Underwood, A. P., Sakuda, S., and Bianco, A. E. (2001). Expression and secretion of a larval-specific chitinase (family 18 glycosyl hydrolase) by the infective stages of the parasitic nematode, *Onchocerca volvulus*. J. Biol. Chem. 276(45):42557-64.

Xiao, Y., Liu, T. T., Dai, H., Zhang, J. J., Liu, H., Tang, H., Leak, D. J., and Zhou, N. Y. (2012). OnpA, an unusual flavin-dependent monooxygenase containing a cytochrome b (5) domain. J. Bacteriol. 194(6):1342-9.

Yang, K., Liu, Y., Liang, L., Li, Z., Qin, Q., Nie, X., and Wang, S. (2017). The high-affinity phosphodiesterase PdeH regulates development and aflatoxin biosynthesis in *Aspergillus flavus*. Fungal. Genet. Biol. 101:7-19.

Yang, Y., Lu, J., Sun, B. J., Wang, D., Yin, X.Y., and Ren, D. F. (2011). Inhibitory kinetics study of resveratrol on tyrosinase activity in vitro. J. Microbiol. Biotechn. 30(4):632-635.

Ye, G.J., Breslow, E.B., and Meister, A. (1996). The amino acid sequence of rat kidney 5-oxo-L-prolinase determined by cDNA cloning. J. Biol. Chem. 1(50):32293-300.

Young, C. A., Bryant, M. K., Christensen, M. J., Tapper, B. A., Bryan, G. T., and Scott, B. (2005). Molecular cloning and genetic analysis of a symbiosis-expressed gene cluster for lolitrem biosynthesis from a mutualistic endophyte of perennial ryegrass. Mol. Genet. Genomics. 274(1):13-29.

Zhan, M., Usman, I. M., Sun, L., and Kanwar, Y. S. (2015). Disruption of renal tubular mitochondrial quality control by Myo-inositol oxygenase in diabetic kidney disease. J. Am. Soc. Nephrol. 26(6):1304-21.

Zhang, W. W., Chan, K. F., Song, Z., and Matlashewski, G. (2011). Expression of a *Leishmania donovani* nucleotide sugar transporter in *Leishmania major* enhances survival in visceral organs. Exp. Parasitol. 129(4):337-45.

Zhang, Y., Su, C., Lei, J., Chen, L., Hu, H., Zeng, S., and Yu, L. (2019). Studies on the L-2-hydroxy-acid oxidase 2 catalyzed metabolism of S-mandelic acid and its analogues. Drug. Metab. Pharmacokinet. 34(3):187-193.

Zhou, S., Fujimuro, M., Hsieh, J. J., Chen, L., Miyamoto, A., Weinmaster, G., and Hayward, S. D.

(2000). SKIP, a CBF1-associated protein, interacts with the ankyrin repeat domain of NotchIC to facilitate NotchIC function. Mol. Cell. Bi

TABLE S3 Verification of DGE results by qRT-PCR analysis

Gene ID	Gene product description	Primers (5′-3′)
MAC_08744	SpoC1-C1C	TCTTCCTGGACTTGTGAT<br>GAGCGGTAATGGTAATGG
MAC_07294	Cysteine rich protein	GATTCAACGATATGGCAAAT<br>ATAGGTATCACGGATGTAGA
MAC_05691	Major facilitator superfamily domain containing protein 5	GCTTGCCGATATTATTCCT<br>GCCTGTAAACCTCTTTCAT
MAC_06615	Putative cyclic nucleotide gated channel beta 1	AATACCCGTCGCATTATAC<br>ACTCATCTGTAGCAAGGA
MAC_02204	Cell wall protein	TACGGCTTTCTTGATACA<br>TTGCTGAGTAATCTTGTTG
MAC_08696	Endo-N-acetyl-beta-D-glucosaminidase precursor	CCATCTCAATGCTACCAT<br>AGGAGGATAGTCGTTCAA
MAC_08095	Amidohydrolase family protein	CCTTGTTTGCCAGTTATG<br>GAGTCCTTGTGATTGATG
MAC_07120	Cytochrome P450 52A11	CGGTTGAAGGTCGTATTG<br>TCGGTTGTGAAGATGCTA
MAC_07387	4-coumarate-CoA ligase 2	CCCGTTATTGACATTTGG<br>TCGTTGAAGGTGTATGAG
MAC_01566	MFS monosaccharide transporter, putative	GCTTGATTACTCCTCCTAT<br>CTTGAACAGATCGTCCAT
MAC_05133	Cell wall protein	TATACGACAACATTGACGAAT<br>CGATGGGAGGTGTAATCT
MAC_02205	Chitinase 18-15	CCTATCTTCCCATCATCAA<br>TGAATCTCCTGAACATCC
MAC_07957	Lactate dehydrogenase	TATACCGCAATGTGTCCAT<br>CAGAGTCGTAGCCAAGTC
MAC_04483	Proline oxidase Put1, putative	AAGTGCGATTGTTAATAGATG<br>TTCAGGTAGGCTTGGTAT
MAC_05794	3,2-trans-enoyl-CoA isomerase precursor, putative	CCAAGTTCTACTCCAACGG<br>TACGACTTGCGTCAGGAG
MAC_08428	NDT80 / PhoG like DNA-binding family protein	ATATAGACGAGTATCAGATC<br>TGTGTAACCTGTCAATGG
MAC_01513	RTA1 domain protein, putative	ATCTCACTTCTTGCCATATAC<br>CGCTACCACCATCAATAC
MAC_09183	Dihydrofolate reductase	GACACATTCTTTCCTCTG<br>GCAATTCCATCCTCAATC

MAC_09507	Hydrophobin	TCTTGTTCTCGGTGTTAT<br>TTATGGTGGTGTCAGTAC
MAC_03493	Nitrite reductase	AAGGGCGTATTTGTCTAT<br>CTCTCAAGGTCCATCATT
MAC_04470	Catalase	TTCTACCTCGCTCACTGA<br>TATGCTATGAAGGAGTTGTT
MAC_01848	Indole-diterpene biosynthesis protein PaxU	ACGACAAGTCCACATATC<br>GGAATACGACATGAGTGA
MAC_07816	Inorganic pyrophosphatase	TAACTTCGTGGTTGAGAT<br>CTTCTTCTTTGAGGTATCG
MAC_09373	A/G-specific adenine glycosylase	GTGAATCTCCTAAGCATAT<br>ATCTCCTATTCCACACTC
